# Risk Assessment of Phthalates and Their Metabolites in Hospitalized Patients: A Focus on Di- and Mono-(2-ethylhexyl) Phthalates Exposure from Intravenous Plastic Bags

**DOI:** 10.3390/toxics10070357

**Published:** 2022-06-30

**Authors:** Yolande Saab, Emilia Oueis, Stephanie Mehanna, Zahi Nakad, Rita Stephan, Rony S. Khnayzer

**Affiliations:** 1 Pharmaceutical Sciences Department, School of Pharmacy, Lebanese American University, Chouran, Beirut 1102-2801, Lebanon; emilia.oueis@ku.ac.ae (E.O.); rita.stephan@lau.edu.lb (R.S.); 2 Department of Natural Sciences, Lebanese American University, Chouran, Beirut 1102-2801, Lebanon; stephanie.mehanna@lau.edu.lb; 3 Electrical and Computer Engineering Department, School of Engineering, Lebanese American University, Chouran, Beirut 1102-2801, Lebanon; zahi.nakad@lau.edu.lb

**Keywords:** phthalates, di-(2-ethylhexyl) phthalate, mono-(2-ethylhexyl) phthalate, IV bags, GC–MS, toxicities

## Abstract

Phthalate esters (PAEs) are plasticizers associated with multiple toxicities; however, no strict regulations have been implemented to restrict their use in medical applications in Lebanon. Our study aimed at assessing the potential risks correlated with phthalate exposure from IV bags manufactured in Lebanon. GC–MS analysis showed that di-(2-ethylhexyl) phthalate (DEHP) is the predominant phthalate found in almost all samples tested with values ranging from 32.8 to 39.7% *w*/*w* of plastic. DEHP concentrations in the IV solutions reached up to 148 µg/L, as measured by SPME-GC–MS/MS, thus resulting in hazard quotients greater than 1, specifically in neonates. The toxicity of DEHP is mainly attributed to its metabolites, most importantly mono-(2-ethylhexyl) phthalate (MEHP). The IV bag solution with the highest content in DEHP was therefore used to extrapolate the amounts of urinary MEHP. The highest concentrations were found in neonates having the lowest body weight, which is concerning, knowing the adverse effects of MEHP in infants. Our study suggests that the use of IV bags manufactured in Lebanon could pose a significant risk in hospitalized patients, especially infants in neonatal care. Therefore, Lebanon, as well as other countries, should start imposing laws that restrict the use of phthalates in medical IV bags and substitute them with less toxic plasticizers.

## 1. Introduction

Plasticizers are added during the manufacturing of plastics to increase plasticity and optimize durability [1]. Phthalate esters (PAEs) are among the most widely used plasticizers, especially in the production of poly-vinyl chloride (PVC), the plastic of choice in multiple sectors and applications, such as food packaging, tubing and piping, flooring, wiring and cables, medical products, etc. [2]. PVC thermoplastic accounts for about 40% of the plastic material used in the medical device industry [3]. It is a main constituent of IV bags and tubing but is also used for a wide range of other healthcare applications, such as oxygen masks, catheters, nasal cannulas, dialysis equipment, ostomy bags, etc. [4].

It is known that phthalate plasticizers detach and leach from the plastic matrix since they are not covalently bound to it; PAEs either dissolve in liquids and fats or spread in the ambient air [4]. Hence, human exposure to PAEs comes from various direct and indirect sources and occurs through the inhalation of contaminated air, ingestion of contaminated food, and skin contact [5]. Another noticeable route of exposure is through medical devices [6]. Di-(2-ethylhexyl) phthalate (DEHP) is the most prominent phthalate plasticizer and constitutes on average about 20–40% by weight of the plastic medical device [7]. Even though DEHP is the most commonly used for PVC, other PAEs are sometimes included in PVC and non-PVC medical devices, such as diethyl phthalate (DEP), benzyl butyl phthalate (BBP), and diisobutyl phthalate (DIBP), to name but a few [8].

PAEs in general and DEHP in particular are suspected as endocrine disruptors and there is great concern about their toxicity [4,7]. Some studies have suggested that PAEs contribute to many adverse effects on animal and human health, including infertility [9,10,11], mutagenic activity, and carcinogenicity [12]. Exposure to PAEs during pregnancy has also been linked to complications, such as pregnancy loss, premature birth, and altered neurodevelopment [13,14,15]. Furthermore, childrens’ exposure to PAEs might potentially result in an increased risk of allergic diseases, including asthma and eczema, as well as alterations in physical development, social behavior, and cognitive development [16,17].

In the body, phthalates are metabolized in two phases before elimination via urine (primary excretion route), feces, and sweat. In the first phase, the ester bonds are hydrolyzed to monoesters by lipase or esterase, and in the second phase, phthalates are converted into sulpho- or glucuro-conjugates [18,19,20]. Epidemiological studies have confirmed that phthalate metabolites (mPAEs) are more toxic than their parent phthalate, based on their pharmacokinetic properties [21,22]. Mono(2-ethylhexyl) phthalate (MEHP) is one of the primary metabolites of DEHP; it is excreted in urine within 48 h after exposure to the phthalate in humans [19]. MEHP was shown to alter the expression of hypothalamic–pituitary–thyroid (HPT) genes, thus disrupting the thyroid endocrine system in zebrafish larvae [4]. A study on Chinese children found a positive correlation between MEHP exposure and obesity, as evidenced by high BMI and waist circumference [23]. A similar association was reported with the exposure to mono(2-ethyl-5-oxohexyl) phthalate (MEOHP) and mono(2-ethyl-5-hydroxyhexyl) phthalate (MEHHP) metabolites in adult males [24]. Acute testicular toxicity was also observed after short-term exposure of Wistar rats to MEHP, which triggered the breakdown of the vimentin filament in Sertoli cells as well as the separation and shedding of germ cells [25]. This effect was not observed in humans, possibly because rodents and humans have different steroidogenesis pathways [26].

As DEHP was classified as a hazardous compound, it was banned or restricted to 0.1% (*w*/*w*) in toys and childcare articles by the European Union [Directive 2005/84/EC] and the Consumer Product Safety Improvement Act as of February 2009 in the US [27]. Amendments on Entry 51 of Annex XVII to REACH Regulation (EC) No 1907/2006 expanded the scope to any plasticized materials in articles [28]. However, several items were excluded from these restrictions, including medical devices, where DEHP and other phthalates are still used, even though the European scientific committee on emerging and newly identified health risks (SCENIHR) [29,30] encourages the use of alternative plasticizers, if available. The latter are replacing DEHP and phthalates in numerous applications, not only childcare products—most notably medical devices—and they include di-2-ethylhexyl terephthalate (DEHT), diisononyl cyclohexane-1,2-dicarbonylate (DINCH), trioctyl trimellitate or tri-2-ethylhexyl trimellitate (TOTM), di(2-ethylhexyl) adipate (DEHA), and acetyl-tri-*n*-butyl citrate ATBC [31,32]. Alternative plasticizers are considered to be of lower risk for humans even though toxicological data is still limited [33]. Besides DEHP, other phthalates, such as DBP, BBP, DIBP, Di-isononyl phthalate (DINP), Di-isodecyl phthalate (DIDP), and Di-*n*-octyl phthalate (DNOP), are also restricted in toys and childcare articles, under Entries 51 and 52 of Annex XVII to REACH Regulation (EC) No 1907/2006 [28]. Additionally, only 1000 ppm of DBP, BBP, DINP, or DIDP is allowed in sandals and flats, as well as squeegee items, according to the California Proposition 65 [34].

Lebanon does not have any legal regulations concerning the controlled use of phthalate plasticizers in plastic medical device applications [35,36,37]. Therefore, in this study, we assessed whether IV bags manufactured in Lebanon contain phthalates and we performed a quantitative analysis to evaluate the exposure risk. The phthalate content of the bag was determined by GC–MS and the leached amount was measured using solid phase micro-extraction SPME-GC–MS/MS. Based on the data found for DEHP, we conduct a risk assessment analysis using the projected urinary concentrations of the MEHP metabolite according to different weight groups. This is of significance, knowing the toxic activity of MEHP. To our knowledge, no such study has previously been conducted to analyze phthalate exposure from IV bag plastics and solutions that are locally produced.

## 2. Materials and Methods

### 2.1. Chemicals

Di-(2-ethylhexyl) phthalate (DEHP; 99.7%), bis-2-ethylhexyl adipate (DEHA; 99%), and triphenyl phosphate (TPP) were purchased from Sigma-Aldrich. Benzyl butyl phthalate (BBP; 98%), was purchased from Supelco, and phthalate esters mixture (1 mL of 500 µg/mL each in methanol), containing dimethyl phthalate (DMP), diethyl phthalate (DEP), dibutyl phthalate (DBP), BBP, DEHA, and DEHP, was purchased from Sigma-Aldrich, Supelco. Tetrahydrofuran (THF), ethanol, and hexane were purchased from Sigma-Aldrich (MO, USA).

### 2.2. Sample Collection

IV bags from two different local manufacturers were purchased from local pharmacies. Samples’ specifications, including composition and volume, as well as production and expiry dates are presented in Table S1. An intraperitoneal bag manufactured in Europe was analyzed for comparison.

### 2.3. Extraction Procedures

During the whole procedure, care was taken to avoid the usage of gloves, plastic material, and contaminated glass material. All glassware was cleaned with soap, rinsed with water followed by acetone, and then left to dry in a furnace at 120 °C.

#### 2.3.1. Plastic IV Bags for GC–MS Analysis

After emptying the IV solution into a glass container, 0.1 g of the IV bag was cut into small pieces, which were placed in a glass beaker and 25 mL of THF was added. The solubilized solution was covered and stirred at room temperature for 40 min. An amount of 1 mL of TPP at 5 g/L was added as an internal standard to obtain a final concentration of 50 mg/L in the undiluted solution and 5 mg/L in the analyzed solution. An amount of 74 mL of hexane was then added to the mixture to precipitate the PVC. The solution was then centrifuged in a Teflon tube at 3000 rpm for 15 min. The supernatant was diluted 10 fold and the resulting solution was filtered and directly injected onto GC–MS for analysis of DMP, DEP, DBP, BBP, DEHA, and DEHP.

#### 2.3.2. IV Bag Solutions for SPME-GC–MS/MS Analysis

Taking the precautions mentioned above, the IV solutions were emptied and kept in closed glass containers at room temperature. An amount of 2.5 mL of the solutions was added to the vials and directly analyzed using an SPME injector onto GC–MS/MS.

### 2.4. Method Validation

#### 2.4.1. Calibration Curve

GC–MS analysis: The PAEs mixture (0.5 g/L) was used to generate the calibration curves for all five phthalates components (DMP, DEP, DBP, BBP, and DEHP), in addition to DEHA, by GC–MS using TPP as an internal standard. Two intermediate solutions at 50 and 100 mg/L were used to generate the final concentrations used to build the calibration curve in triplicates (0.5, 1.5, 2.5, 5, 15, and 32 mg/L) with the addition of TPP as an internal standard to a concentration of 5 mg/L.

SPME-GC–MS/MS analysis: The PAEs mixture at 0.5 g/L was used to generate the calibration curves for all six components (DMP, DEP, DBP, BBP, DEHA, and DEHP). Two intermediate solutions at 500 and 50 μg/L were used to generate the final concentrations (2.5 mL) used to build the calibration curve (1.5, 2.5, 5, 10, 15, 25, 50, 100, and 500 μg/L) using either a 0.9% NaCl solution or S21-A.

#### 2.4.2. Limit of Detection and Limit of Quantification

The limit of detection (LOD) and limit of quantification (LOQ) for each plasticizer were calculated using the blank (Blk) and low concentration samples (LCS) method [38,39]. The limit of blank (LOB) was first determined by running 10 samples of blank not expected to have any analyte, as described in Equation (1).

LOB = mean_Blk_ + 1.645 × SD_BLK_ (1)

The constant “1.645” is a correction for the biased estimate of the population SD due to sample size [40].

After calculating LOB, LOD can be calculated using the statistical standard deviation of 10 samples at low concentration (LCS), according to Equation (2).

LOD = LOB + 1.645 × SD_LCS_ (2)

The LOQ can further be determined using LOD value, as shown in Equation (3):

LOQ = 3 × LOD(3)

Experimentally, 9 blank solutions were prepared by following the same procedure (extraction, dilution, and analysis) as the actual samples. The average concentration mean_Blk_ and the SD_BLK_ of all samples were considered for determining LOB. Another set of 10 solutions of each plasticizer at a low concentration (0.025 μg/L for DMP, DEP, DBP, BBP, DEHA, and DEHP) was analyzed to determine LOD and LOQ (Tables S3 and S4).

#### 2.4.3. Instrumental Analysis

Analytical GC–MS was performed following a modified procedure [41] on a Schimadzu Nexis GC-2030 GC instrument coupled to a Schimadzu QP2020-NX mass spectrometer using an Agilent DB-5ms column (30 m × 0.25 mm ID × 0.25 µm) by direct liquid injection. The GC method and gradient were optimized to obtain good separation of all six PAEs and the internal standard TPP. The GC flow was directly injected to the mass spectrometer where mass spectra were acquired in the single ion monitoring (SIM) mode. The chromatographic system used and the GC–MS parameters are detailed in the SI.

Analytical SPME-GC–MS/MS was performed on a Thermo Scientific Trace 1310 GC instrument coupled to a Thermo Scientific TSQ 9000 triple quadrupole mass spectrometer using a Thermo Scientific TR-5MS column (30 m × 0.25 mm ID × 0.25 µm) by immersed solid phase micro-extraction. The sample was homogenized continuously during extraction by intermittent agitation in 10 s cycles. The GC method and gradient were optimized to obtain good separation of all six PAEs. The GC flow was directly injected to the mass spectrometer where mass spectra were acquired in the selected reaction monitoring (SRM) mode. The chromatographic system used and the GC–MS/MS parameters are detailed in the SI.

### 2.5. Analysis of Data

#### 2.5.1. Content Calculation

The level of plasticizers in the PVC IV bags was reported in a weight to weight percentage (% *w*/*w*). The direct measurements by MS using the calibration curves gave a concentration of the corresponding plasticizer in the analyzed sample (PAE_i_ in mg/L). The amount of plasticizer extracted from the PVC IV bag was calculated by multiplying the initial extraction volume V_Ex_ of 100 mL (25 mL THF, 74 mL hexane, and 1 mL of TPP) and the resulting concentration PAE_i_ by the dilution factor (DF = 10), Equation (4). The ratio % (*w*/*w*) of plasticizer present in the bag can be calculated by dividing the mass of plasticizer obtained *m_PAE_* by the mass of the plastic used *m_p_*, as shown in Equation (5).

*m_PAE_* = PAE_i_ × DF × V_Ex_ (4)

(5) $$\mathrm{ratio}(\%\;w/w)=\frac{m_{PAE}}{m_{P}}\;\times 100$$

#### 2.5.2. Risk Assessment for Phthalate Exposure and Hazard Index

The amounts of plasticizers were calculated using the calibration curves in both the IV bags and IV solutions. The latter represents the leached amount of plasticizer from the plastic IV bags into the solution, which would eventually be injected intravenously into patients. The toxicity in this case is related to the amount of all the phthalate esters present in the solution, even though each phthalate has a different toxicity level. Using the reference dose (RfD) for each phthalate, which is the recommended maximal exposure per day (µg/kg/day), estimated by the US EPA, a conversion factor (*CF*) was calculated based on the most toxic compound with the lowest RfD, DEHP in this case (Table S2). The *CF* represents the equivalent dose of each phthalate to have the same degree of toxicity as DEHP. The average total amount of leached phthalates (*PAE_T_*) in IV solutions was calculated by summing up the calculated amount of each phthalate multiplied by its corresponding *CF*, as described in Equation (6). Average values from the different IV bags were used for these calculations.

(6) $$PAE_{T}=\overset{n}{\underset{i}{\sum }}PAE_{n}\;x\;CF_{n}$$

To assess whether the amount of phthalate esters, specifically DEHP, leached into IV solutions could represent a health risk, the estimated amount of DEHP injected per hospitalization day (EDI in µg/day) was calculated by multiplying the volume of IV injection (IV_V_) with the total amount of leached phthalates (*PAE_T_*), as shown in Equation (7).

EDI_DEHP_ = *PAE_T_* × IV_V_ (7)

where EDI_DEHP_ is the estimated daily intake per hospitalization of DEHP injected via IV (µg/day) and IVv = 0.5 L/day for neonates or 2.5 L/day for adults.

This number was then divided by the average body weight (BW) per target group (neonates: 1, 2, 3, 4, and 5 kgs; adults: 50, 60, 70, 80, and 90 kgs) to give EDI_W_ (µg/kg/day), as shown in Equation (8).

EDI_W_ = EDI_DEHP_/BW (8)

The hazard quotient (HQ) was determined using Equation (9) below:

HQ = EDI_W_/RfD_DEHP_ (9)

A HQ of one or less indicates that the exposure is equal or less then the allowed RfD level, which indicates that adverse effects are unlikely to occur, considering only this route of exposure.

#### 2.5.3. Risk Assessment for Simulated MEHP Exposure

Since MEHP is one of the putative toxic metabolites of DEHP, data from the latter were used to estimate the urinary concentrations of MEHP.

Equation (10) was proposed by Zang and colleagues to calculate the amount of daily intake of MEHP from the amount found in urinary samples [42].

(10) $$\mathrm{EDI}\left(\mu g/\mathrm{kg}\;\mathrm{bw}/\mathrm{day}\right)=\frac{UE\left(\mu mol/L\right)\times UV\left(L/kg\;bw/day\right)}{F_{UE}}\times MW_{phtalate}\left(g/mol\right)\;$$

*EDI* is the estimated daily intake, *UE* is the urinary excretion, *MW_phthalate_* is the molecular weight of the parent phthalate (DEHP in this case), *UV* is the daily human excretion volume of urine, and *F_UE_* is the “molar fraction of the urinary excreted monoester related to the ingested diester”. The authors suggested a *F_UE_* of (5.9–6.2%) for MEHP based on the literature [43,44].

The main approach is to use Equation (10) to estimate UE using the *EDI* (Equation (11)):

(11) $$\mathrm{UE}=\frac{EDI}{MW}\times \frac{F_{UE}}{UV}$$

The normal urine output is 0.5 to 1.5 mL/kg/h [45], based on which *UV* was determined for the same body weight groups used for *EDI*; neonates (1, 2, 3, 4, and 5 kg) and adults (50, 60, 70, 80, and 90 kg).

Thus, for the final calculation of UE, a range for *F_UE_* and a range for *UV* were obtained; $\frac{EDI}{MW}\times \frac{F_{UEmin}}{UVmax}$ was used to find the minimum value of UE and $\frac{EDI}{MW}\times \frac{F_{UEmax}}{UVmin}$ was used to find the maximum value of UE.

## 3. Results

### 3.1. GC–MS Analysis of IV Bags

The LOD and LOQ values of DEP and DEHP were determined using the method of LOB and LCS described above and were found to be 0.258 mg/L and 0.774 mg/L for DEP and 0.732 and 2.20 mg/L for DEHP, respectively. The other four phthalates were not detected in the blank sample at all, and their values were determined using the same method for LOD and LOQ, where LOB is considered as zero. LOD values varied between 0.013 and 0.035 mg/L and LOQ between 0.038 and 0.106 mg/L. The calibration curves were constructed using 0.5 mg/L as the lowest concentration point, and 32 mg/L as the highest, with a coefficient of determination (R^2^) higher than 0.97 for all phthalates. Data for the calibration curves and LOD/LOQ are presented in Table S3.

After analyzing the samples in triplicates, it was observed that all samples except for one, had considerable amounts of DEHP (Table S5). The amount of DEHP in the samples ranged from 32.8 to 39.7% *w*/*w* by weight of plastic, including the plastic bag manufactured in Europe (Sample 10), which has 34.4% DEHP by mass. Sample 1 showed only traces of DEHP similar to blank levels. The relative standard deviation is below 3.5% for all measured samples. Only trace levels of some of the other phthalates were detected in some samples.

### 3.2. SPME-GC–MS/MS Analysis of IV Solutions

The content of DEHP and other known PAEs in the IV solutions were quantitatively assessed by SPME-GC–MS/MS analysis in duplicates. The first calibration curve was built using a solution of 0.9% NaCl in ultra-high pure water. However, given the very high background level in phthalate esters of the blank solution, and since we were working down to ppb levels, new calibration curves were constructed using the solution from the DEHP-free plastic bag (Sample 1), where no DEHP was expected to be present. The calibration curves were built from 2.5 to 500 μg/L, with a coefficient of determination (R^2^) above 0.94 for all quantification masses of the different phthalates (except two for DEHA that had a value of 0.85). Data for the calibration curves are presented in Table S4. The PAE concentration was determined as an average of the respective three fragment ions for each parent PAE ion (Table S6).

**Figure 1:**
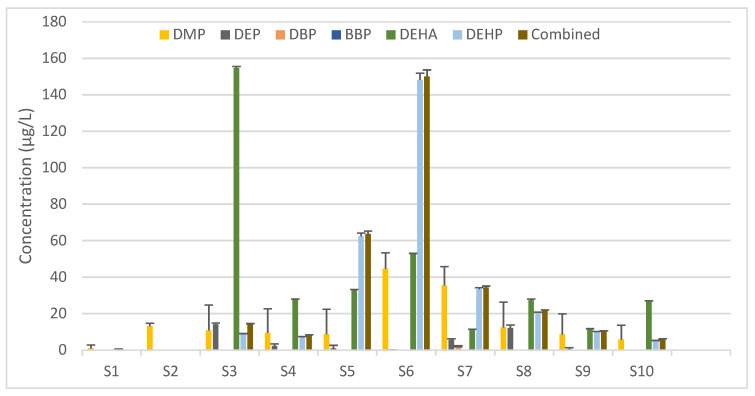
Amount of phthalate ester plasticizers detected in the IV solutions of the analyzed samples and the combined DEHP equivalent concentration for each. Values represent the mean concentration + SD (in µg/L) calculated based on three quantitative ions from duplicates of each sample. DMP, dimethyl phthalate; DEP, diethyl phthalate; DBP, dibutyl phthalate; BBP, benzyl butyl phthalate; DEHA, di-ethylhexyl adipate; DEHP, di-(2-ethylhexyl) phthalate.

The analysis of the IV solutions by SPME-GC–MS/MS showed a significant difference in the amount of DEHP leached in the different samples. Other than the blank (Sample 1), one sample (Sample 2) has no detected DEHP, while the other ones vary from about 4.95 to 148 µg/L with a relative standard deviation less than 3.7% for each sample. Other phthalates were also detected in the IV solutions. DMP was present in all samples with a concentration ranging from 1.03 to 44.5 µg/L. DEP was present in four samples only with concentrations between 0.0390 and 14.0 µg/L. DEHA was present in all but one sample other than the blank, with concentrations varying significantly from 0.226 to 155 µg/L (Figure 1, Table S7). However, the total PAE concentrations obtained using the conversion factors (Table S2) were not significantly different from the DEHP data. Hence, the latter were used for risk assessment.

### 3.3. Risk Assessment for Phthalate Exposure

The DEHP concentrations obtained by SPME-GC–MS/MS analysis were used to estimate the daily exposure for each age/weight group and conclude the associated risk based on the hazard quotients (HQs) obtained. As shown in Table 1, the calculated HQs for Sample 3 are greater than 1 in neonates weighing 1, 2, or 3 kg. Sample 5 also presented an HQ > 1 when assessing the risk in neonates weighing 1 kg.

### 3.4. Risk Assessment for MEHP Exposure

The toxicity of DEHP is attributed to its metabolites, most importantly the monoester phthalate MEHP. Although the simulated daily intakes of DEHP from most samples analyzed were below the reference doses, it is of significance to assess the risk associated with extrapolated urinary MEHP concentrations. The latter simulation was conducted based on the data obtained for Sample 3, which exhibited the highest exposure risk, namely in neonates. Equation (10) was used to compute the urinary MEHP concentrations considering both the minimum F_UE_ (5.9%) and the maximum F_UE_ (6.2%). As shown in Table 2, the neonate group would produce urine with the highest concentration of MEHP, ranging from 10.9 to 273 ng/mL in 5 kg and 1 kg babies, respectively, at the maximum F_UE_. In adults, concentrations range from 0.168 to 0.546 ng/mL in 90 kgs and 50 kgs individuals, respectively.

## 4. Discussion

According to our results, DEHP is the single most used plasticizer in plastic for IV bags manufactured in Lebanon. The amount of DEHP ranged from 32.8 to 39.7% by weight with an average of 35.4%. This value is consistent with the usual amounts of DEHP (20–40%) used for medical devices [7]. Only one sample was DEHP-free (Sample 1) but we could not find any indication as to the difference in the plastic used from the same manufacturer of other samples. An intraperitoneal bag manufactured in the EU was also assessed and found to contain similar amounts of DEHP (34.4%). Other plasticizers were detected only in trace levels in the plastic and could either have been used in very small amounts or been present as contaminants during the production and processing of the plastic. Given the absence of any local or international regulation banning the use of DEHP-containing plastic in medical devices, at the very least, an indication of the presence of such compounds, which are classified as carcinogenic, mutagenic, or endocrine disruptors (category 1 or 2), would be expected. There was no indication on the IV bags to note that DEHP was used as a plasticizer. The brand name of the type of plastic used was only indicated on the IV bag. One local manufacturer uses PVC PL-146 plastic, which contains DEHP, as stated by the plastic manufacturing company. This explains the high DEHP content of the IV bags observed. The other local manufacturer specifies a different brand name, information on which could not be found. A European directive [46] indicates that medical devices containing phthalates which are classified as category 1 or 2 must be labelled. However, the tested intraperitoneal infusion bag manufactured in a country within the European Union contained similar amounts of DEHP (34.4%) and lacked any labelling pertaining to its presence. As this sample was purchased locally and was clearly meant for distribution outside the European Union (including description in the Arabic language and registration numbers in two Arabic countries), it is highly likely that European regulations were not followed.

A recent study was conducted in two academic hospitals in Belgium and the Netherlands to identify plasticizers in indwelling plastic medical devices commonly used in the pediatric intensive care unit (PICU) [47]. It was found that about 60% of the samples contained DEHP as the major plasticizer, followed by DEHA, bis(2-ethylhexyl) terephthalate (DEHT), TOTM, ATBC, and others. Some showed the presence of more than one plasticizer, most notably the presence of DEHP and DEHT with TOTM. DEHP and DEHT are believed to be common contaminants in the production of TOTM [47,48]. This result comes into stark contrast with the European recommendations. France is the only country so far to limit the use of DEHP plasticizers to 1% in medical tubing intended for use in pediatrics, neonatology, and maternity wards. Nonetheless, this law can be disregarded when no known safer replacements can be applied.

Studies have shown that DEHP has the highest migration abilities [31,49,50], especially in medical equipment using PVC [8], which explains why the amounts of DEHP detected in some of the IV solutions go up to 148 ppb. The plastic PL-146 is claimed to leach a maximum of 5 ppm (5 mg/L) into the solution when close to its expiration date. The amounts we observed range from 6.6 to 148 ppb with a high variability between the samples. Previous studies have shown DEHP values in various IV crystalline solutions (normal saline, glucose, Ringers, etc.) ranging between 0.22 and 34.00 μg/L depending on the different sample, their lifetime, and their composition [7,51,52,53]. One other study we could find showed a much higher concentration of 1.04 mg/L of DEHP in a 0.9% saline solution [8]. This high discrepancy in the results could be due to the method of extraction used, the individual samples transportation, and storage. Far higher amounts of DEHP have been found in other types of solutions, including total parenteral nutrition (TPN) with lipids (230–450 μg/L) [54], and a number of drug containing solutions, such as paclitaxel (6600–56,600 μg/L) and etoposide (17,000–25,000 μg/L), as it has been shown that more lipophilic solutions increase the migration of plasticizers and that certain types of drugs adhere to the plastic surface [55,56,57,58].

DEHA and DMP were found in almost all samples, whereas DEP and DBP were present in fewer samples at lower concentrations, and BBP was completely absent. As none of these other phthalates were detected in the IV plastic bags, their origin could not be accurately determined. These could have still originated from the plastic IV where all traces would have leached from contamination during the manufacturing process not pertaining to the plastic bags or from contaminated starting materials of the IV composition.

In our analysis, we found that DEHP exposure from the high concentration IV infusions results in levels above RfD reference in neonates, specifically those with the lowest weight range, including premature babies. Monitoring studies have actually shown that phthalate exposure in children is significantly higher compared to the adult population [59]. Owing to their small size and exposure to multiple invasive medical procedures, premature infants exhibit phthalate levels that are over 4000 times higher than the safe dose for reproductive toxicity [60]. Jenkins et al. reported a positive correlation between hypertension and DEHP exposure in premature babies where there was evidence of 11β-HSD_2_ inhibition and increased activity of the sodium channel [61]. Additionally, Von Rettberg and colleagues reported a 5.6-fold increase in the risk of cholestasis when neonates received parenteral food via DEHP-plasticized PVC tubing compared to PVC-free tubing [62]. Other studies have also linked prenatal DEHP exposure with impaired cognitive functions and motor control, as well as executive abilities [63,64,65,66].

Assessing the risk associated with phthalate exposure using DEHP-based quantification can be misleading because of potential contamination from laboratory equipment, which includes phthalate diesters [67]. The latter are quickly metabolized to their phthalate monoesters and is excreted primarily in urine [20]. Owing to their high stability, lack of contamination in laboratory settings, low detection limits upon analysis, and higher toxicity compared to the parent phthalate, urinary metabolites are selected in most studies as biomarkers for phthalate exposure and related adverse effects [68]. Our simulation has shown that exposure to Sample 3, which exhibited the highest DEHP concentrations and HQ > 1, would result in urinary MEHP concentrations that are particularly elevated in the neonate group, reaching a maximum of 273 ng/mL in 1 kg infants. This number is 2.73-fold higher than the mean MEHP concentration measured by Calafat and colleagues in urine samples retrieved from premature newborns [69] and over 18 times greater than the average value reported by Weuve et al. in neonates [70]. Infants likely have a different DEHP metabolism compared to older children or adults, including low glucuronidation activity, which may increase the half-life of MEHP and delay the excretion of DEHP [71], as well as high levels of gastric lipases, which are involved in the metabolism of DEHP to MEHP [70]. MEHP has been associated with a number of adverse effects in neonates, such as the inhibition of neutrophil migration and apoptosis [72].

Although the urinary MEHP levels we obtained in the adult group are lower than the reported concentrations associated with different toxicities [20,23,67,73], our analysis accounts for only one source of phthalate exposure (IV bags), which would constitute only a portion of the total exposure.

Another vulnerable group of patients are those with decreased kidney function. With the higher levels of DEHP and longer hospitalization stays requiring daily infusions, DEHP and its metabolites levels and accumulation quickly rise because of reduced excretion, making it a huge contributor of phthalate exposure in addition to other routes. As for patients with chronic diseases and drug intake, another concern is the risk of interaction between the DEHP–MEHP metabolism and patients’ polypharmacy.

High levels of DEHP exposure in pregnant women have been shown to affect both the pregnancy outcome and the fetus [74]. Toxicity studies conducted in rats determined the no-observed-adverse-effect level (NOAEL) to be 200 mg/kg per day [75] for maternal and embryonal toxicity. Even though the numerical determination of fetal DEHP levels based on maternal consumption has, to our knowledge, not been studied previously, it remains certain that pregnant women would constitute at high risk groups of patients when subjected to long hospitalization stays, increasing their DEHP and PAE exposure.

It remains to be mentioned that this study focusses mainly on DEHP and PAEs emanating from the injected IV solutions. However, in a normal setting, IV bags would be connected through plastic tubing and stopcocks, which on their own also have considerable amounts of PAEs. Not to mention that drugs added to IV injections also affect and mostly increase the amount of leached PAEs into solution, making drug injections even more problematic. More often than not, neonates in ICU units requiring IV injections are also on respirators connected through PVC tubing, increasing their exposure to PAEs [71]. In addition to medical devices, non-medical sources of PAE exposure should also be taken into account, including ambient air, food in plastic packaging, and any other plastic items that could be around a patient. DEHP or PAEs in IV bags on their own are mostly not toxic, but when considered for at risk groups in addition to the other medical and non-medical sources over a lengthy period of time, the risk could become highly significant. Moreover, it is quite difficult to predict the accurate amount of leached phthalate esters in general and DEHP in particular into the IV solution from the plastic bags, as it seems to be affected by a multitude of factors, some of which were not considered in this study, such as the manufacturing process, storage before and after sale, transportation, and temperature. It was not possible to establish any trend in regard to the composition, shelf life, and DEHP content in the plastics.

## 5. Conclusions

Although the risks associated with the exposure to phthalates are well established, international federations have not enforced clear restrictions as to their use in medical devices like IV bags. This work aimed at assessing the potential risks related to the use of plastic IV bags manufactured in Lebanon and DEHP was found to be the predominant phthalate and its quantification revealed concerning results, especially when assessing the associated risks in neonates. Simulated urinary concentrations of MEHP, a major toxic metabolite of DEHP. The derived numbers were several orders of magnitude higher than the values reported in the literature for the neonate group, which could be an alarming finding. Although the numbers obtained in adults are lower than the reported concentrations associated with different toxicities, our analysis accounts for only one source of phthalate exposure (IV bags), which would constitute only a portion of the total exposure, not to mention that patients with highly reduced kidney function and pregnant women are particularly vulnerable.

Therefore, more research is needed in order to determine the safety profile of any component that might be used in plastics meant for human-use applications, such as medical devices and food storage and packaging. It is worth mentioning that these changes must include all parts of the medical devices and not just the containers or tubing, for example. The effect of the physiological solutions intended for each application would have to be tested as well (nutrients, blood, saline, glucose, drugs, etc.), as it has been shown that more lipophilic solutions increase the migration of plasticizers and that certain types of drugs adhere to the plastic surface. Another strategy used by researchers is the physical and chemical modification of the PVC polymer in order to limit or prevent plasticizer migration.

The effective move towards the use of safer plasticizers in medical equipment requires a large-scale mobilization and collaboration between regulation agencies, expert scientists, manufacturers, and users (most notably hospitals and patient care centers). More research into alternative plasticizers, such as esters of fatty acids, citric acids, as well as other alternatives like di(isononyl)cyclohexane-1,2-dicarboxylate (DINCH), aliphatic polyesters, TOTM, etc., is required before regulators can ensure their enforcement and ban the use of particularly harmful ones (such as DEHP).

## Figures and Tables

**Table 1:** Risk assessment of di-(2-ethylhexyl) phthalate (DEHP) exposure in the analyzed IV-bag solutions. Numbers represent the hazard quotient (HQ) measured using the equation HQ_T_ = EDI_wT_/RfD_DEHP_, where EDI_WT_ is the estimated daily intake by weight and RfD_DEHP_ is the reference dose for DEHP (20 mcg/kg/day).

**Hazard Quotient (HQ)**		**Neonates (kg)**					**Adults (kg)**				
		**1**	**2**	**3**	**4**	**5**	**50**	**60**	**70**	**80**	**90**
	**Sample 1**	0.000	0.000	0.000	0.000	0.000	0.000	0.000	0.000	0.000	0.000
	**Sample 2**	0.000	0.000	0.000	0.000	0.000	0.000	0.000	0.000	0.000	0.000
	**Sample 3**	3.71 *	1.85 *	1.24 *	0.926	0.741	0.371	0.309	0.265	0.232	0.206
	**Sample 4**	0.832	0.416	0.277	0.208	0.166	0.0832	0.0693	0.0594	0.0520	0.0462
	**Sample 5**	1.56 *	0.780	0.520	0.390	0.312	0.156	0.130	0.111	0.0975	0.087
	**Sample 6**	0.221	0.110	0.0740	0.0552	0.0442	0.0221	0.0184	0.0160	0.0138	0.0123
	**Sample 7**	0.172	0.0860	0.0573	0.0429	0.0344	0.0172	0.0143	0.0123	0.0107	0.00954
	**Sample 8**	0.510	0.255	0.170	0.128	0.102	0.0510	0.0425	0.0365	0.0319	0.0284
	**Sample 9**	0.248	0.124	0.0825	0.0619	0.0495	0.0247	0.0206	0.0177	0.0155	0.0138
	**Sample 10**	0.124	0.0619	0.0413	0.0310	0.0248	0.0124	0.0103	0.00885	0.00774	0.00688

* HQ values > 1.

**Table 2:** Estimation of the urinary mono-(2-ethylhexyl) phthalate (MEHP) concentrations in neonate and adult groups (in µg/L) using di-(2-ethylhexyl) phthalate (DEHP) levels obtained for Sample 3.

		UEmin (µg/L)	UEmax (µg/L)
**Neonates (kg)**	1	86.6	273
	2	21.6	68.2
	3	9.62	30.3
	4	5.41	17.1
	5	3.46	10.9
**Adults (kg)**	50	0.173	0.546
	60	0.120	0.379
	70	0.0883	0.278
	80	0.0676	0.213
	90	0.0534	0.168

UE_min_, minimum urinary excretion concentration; UE_max_, maximum urinary excretion concentration.

## Data Availability

Data presented in this study are available in the supplementary material.

## Supplementary Materials

The following supporting information can be downloaded at: https://www.mdpi.com/article/10.3390/toxics10070357/s1. Supplementary Methods: GC-MS parameters used for phthalate quantification in IV bags; SPME-GC-MS/MS parameters used for phthalate quantification in IV bag solutions. Table S1: Specifications of the samples used for analysis. Table S2: Reference doses of the plasticisers analyzed, estimated by the US EPA, and conversion factors based on the most toxic analyte (DEHP). Table S3: GC-MS calibration curves data. Table S4: SPME-GC-MS/MS calibration curves data. Table S5: GC-MS/MS analysis results showing the PAEs content in the IV plastic bags. Table S6: Confir-mation (parent) and quantitative (fragment) ions selected for each analyte, as measured by SPME-GC-MS/MS, with the corresponding retention time and collision energy used. Table S7: SPME-HS-GC-MS/MS analysis results showing the PAEs content in the IV plastic bags. The total amount was computed by summing up the calculated amount of each phthalate multiplied by its corresponding CF (Table S2).
