# Supplementary material for: Risk Assessment of Phthalates and Their Metabolites in Hospitalized Patients: A Focus on Di- and Mono-(2-ethylhexyl) Phthalates Exposure from Intravenous Plastic Bags
Source: Toxics. 2022 Jun 30;10(7):357. doi: 10.3390/toxics10070357 (PMC9324282; doi:10.3390/toxics10070357)
Supplement: Supplementary file 1 [file toxics-10-00357-s001.zip › toxics-1753202-supplementary.pdf]

Supplementary Methods

GC-MS parameters used for phthalate quantification in IV bags

The components were injected by a syringe heated to 40 °C in splitless mode. The inlet was maintained at 220 °C and helium was used as the carrier gas at a flow rate of 1.2 mL.min^{-1}. The oven temperature was initially set at 100 °C for 1 min and increased to 200 °C at 20 °C.min^{-1}, then to 250 °C at 4 °C.min^{-1} (hold time: 2 min) and finally to 280 °C at 20 °C.min^{-1} [1] (hold time: 5 min). The quadrupoles were operated using the following parameters: the MS transfer line was kept at 250 °C and the ion source was kept at 220 °C. Electron impact was used as the ionization mode, and data were acquired between 50 and 420 m/z.

SPME-GC-MS/MS parameters used for phthalate quantification in IV bag solutions

The components were injected by a syringe heated to 40 °C in splitless mode. The inlet was maintained at 280 °C and helium was used as the carrier gas at a flow rate of 1.0 mL.min^{-1}. The oven temperature was initially set at 100 °C for 5 min and increased to 200 °C at 20 °C.min^{-1}, then to 250 °C at 4 °C.min^{-1} (hold time: 2 min) and finally to 280 °C at 20 °C.min^{-1} (hold time: 5 min). The quadrupoles were operated using the following parameters: the MS transfer line was kept at 250 °C and the ion source was kept at 220 °C. Electron impact was used as the ionization mode, and quantification was based on three product ions for each phthalate, as described in Table S6 below.

Table S1 Specifications of the samples used for analysis.

**Sample**	**Manufacturer**	**Composition**	**Volume**	**Production Date**	**Expiration Date**
Sample 1	A	Lactated Ringer's	1 L	02/18	02/21
Sample 2	A	0.9% NaCl	1 L	01/19	01/22
Sample 3	B	5% Dextrose<br>0.45% NaCl	1 L	01/19	01/22
Sample 4	B	5% Dextrose<br>0.9% NaCl	1 L	10/18	10/21
Sample 5	B	0.9% NaCl	1 L	09/18	09/21
Sample 6	A	0.9% NaCl	1 L	07/19	07/22
Sample 7	A	5% Dextrose<br>0.45% NaCl	1 L	07/19	07/22
Sample 8	B	5% Dextrose<br>0.45% NaCl	1 L	01/19	01/22
Sample 9	B	5% Dextrose<br>0.9% NaCl	1 L	10/18	10/21
Sample 10	EU	Intraperitoneal bag	2 L		

Table S2 Reference doses of the plasticisers analyzed, estimated by the US EPA, and conversion factors based on the most toxic analyte (DEHP).

	DMP	DEP	DBP	BBP	DEHA	DEHP
RfD (µg/kg/day)	-	800	100	200	600	20
CF	-	0.025	0.2	0.1	0.033333	1

DMP, dimethyl phthalate; DEP, diethyl phthalate; DBP, dibutyl phthalate; BBP, benzyl butyl phthalate; DEHA, di-ethylhexyl adipate; DEHP, di-(2-ethylhexyl) phthalate.

Table S3 GC-MS calibration curves data.

PAE	LOD (mg/L)	LOQ (mg/L)	Calibration Curve Equation	R^2
DMP	0.035	0.106	y = 6.1466 x - 0.0494	0.9983
DEP	0.258	0.774	y = 7.0211 x - 0.1859	0.9993
DBP	0.024	0.072	y = 7.8253 x + 1.1105	0.9798
BBP	0.013	0.039	y = 2.3091 x - 0.4236	0.9969
DEHA	0.013	0.038	y = 3.0741 x - 0.3912	0.9988
DEHP	0.732	2.20	y = 3.0568 x - 0.4072	0.9983

DMP, dimethyl phthalate; DEP, diethyl phthalate; DBP, dibutyl phthalate; BBP, benzyl butyl phthalate; DEHA, di-ethylhexyl adipate; DEHP, di-(2-ethylhexyl) phthalate.

Table S4 SPME-GC-MS/MS calibration curves data

PAE	LOD (µg/L)	LOQ (µg/L)	Mass Fragment	Calibration Curve Equation	R^2
DMP	0.0541	0.162	77	y = 0.00004 x – 3.095	0.9956
			133	y = 0.00009 x – 6.360	0.9961
			135	y = 0.0001 x – 2.527	0.9958
DEP	0.443	1.33	65.1	y = 0.00002 x – 12.55	0.9965
			121	y = 0.00003 x – 13.82	0.9966
			149	y = 0.00002 x – 12.36	0.9965
DBP	0.612	1.84	65	y = 0.0000005 x – 31.29	0.9954
			93	y = 0.0000008 x – 31.80	0.9956
			121	y = 0.0000008 x – 31.99	0.9951
BBP	0.985	2.95	65	y = 0.000003 x – 2.669	0.9945
			121	y = 0.000005 x – 2.311	0.9944
			149	y = 0.000004 x – 3.032	0.9948
DEHA	0.733	2.20	55.1	y = 0.00002 x – 22.58	0.9979
			83	y = 0.00003 x – 3.483	0.8541
			101	y = 0.00002 x – 3.174	0.8506
DEHP	0.149	0.447	168.1	y = 214924 x + 3000000	0.9507
			168.6	y = 0.000007 x + 0.7615	0.942
			325.3	y = 0.000003 x + 0.6601	0.944

DMP, dimethyl phthalate; DEP, diethyl phthalate; DBP, dibutyl phthalate; BBP, benzyl butyl phthalate; DEHA, di-ethylhexyl adipate; DEHP, di-(2-ethylhexyl) phthalate.

Table S5 GC-MS/MS analysis results showing the PAEs content in the IV plastic bags.

**Sample**	**DMP %w/w**	**SD**	**DEP %w/w**	**SD**	**DBP %w/w**	**SD**	**BBP %w/w**	**SD**	**DEHA %w/w**	**SD**	**DEHP %w/w**	**SD**
Sample 1	0.05	0.0006	0.08	0.0003	-	0.0021	0.00	0.0000	0.00%	0.0000	0.75	0.0002
Sample 2	0.05	0.0006	0.09	0.0005	-	0.0018	0.00	0.0000	0.00%	0.0000	39.70	0.0104
Sample 3	0.04	0.0006	0.10	0.0004	-	0.0019	0.10	0.0014	0.00%	0.0000	34.7	0.0075
Sample 4	0.04	0.0006	0.08	0.0004	-	0.0010	0.10	0.0014	0.00%	0.0000	34.9	0.0077
Sample 5	0.05	0.0006	0.10	0.0004	-	0.0002	0.00	0.0000	0.00%	0.0000	32.8	0.0084
Sample 6	0.04	0.0006	0.10	0.0004	-	0.0011	0.10	0.0014	0.07%	0.0010	38.2	0.0074
Sample 7	0.04	0.0006	0.09	0.0005	-	0.0011	0.00	0.0000	0.00%	0.0000	35.7	0.0095
Sample 8	0.04	0.0006	0.07	0.0004	-	0.0003	0.00	0.0000	0.00%	0.0000	34.5	0.0118
Sample 9	0.04	0.0006	0.09	0.0005	-	0.0002	0.30	0.0042	0.00%	0.0000	32.8	0.0053
Sample 10	0.03	0.0004	0.09	0.0006	-	0.0019	0.00	0.0000	0.00%	0.0000	34.4	0.0048

DMP, dimethyl phthalate; DEP, diethyl phthalate; DBP, dibutyl phthalate; BBP, benzyl butyl phthalate; DEHA, di-ethylhexyl adipate; DEHP, di-(2-ethylhexyl) phthalate.

Table S6 Confirmation (parent) and quantitative (fragment) ions selected for each analyte, as measured by SPME-GC-MS/MS, with the corresponding retention time and collision energy used.

Analyte	Retention Time	Parent ion mass (m/z)	Fragment ion mass (m/z)	Collision Energy (V)
DMP	9.87	163.1	77	20
		163.1	133	8
		163.1	135	10
DEP	10.91	149.1	65.1	20
		149.1	121	10
		177.1	149	8
DBP	14.44	149.1	65	20
		149.1	93	16
		149.1	121	12
BBP	20.31	149	65	20
		149	121	12
		206.2	149	8
DEHA	20.42	129	55.1	14
		129	83	10
		129	101	6
TPP	21.23	215	168.1	16
		326.1	168.6	28
		326.1	325.3	10

DMP, dimethyl phthalate; DEP, diethyl phthalate; DBP, dibutyl phthalate; BBP, benzyl butyl phthalate; DEHA, di-ethylhexyl adipate; DEHP, di-(2-ethylhexyl) phthalate, TPP, triphenyl phosphate.

Table S7 SPME-HS-GC-MS/MS analysis results showing the PAEs content in the IV plastic bags. The total amount was computed by summing up the calculated amount of each phthalate multiplied by its corresponding CF (Table S2).

Sample	DMP (μg/L)	SD	DEP (μg/L)	SD	DBP (μg/L)	SD	BBP (μg/L)	SD	DEHA (μg/L)	SD	DEHP (μg/L)	SD	Total (μg/L)	SD
Sample 1	1.029	1.783	0.000	0.000	0.000	0.000	0.000	0.000	0.226	0.392	0.000	0.000	0.008	0.013
Sample 2	13.066	1.582	0.000	0.000	0.000	0.000	0.000	0.000	0.000	0.000	0.000	0.000	0.000	0.000
Sample 3	10.699	13.956	14.013	0.754	0.000	0.000	0.000	0.000	154.654	0.893	8.838	0.139	14.344	0.112
Sample 4	9.376	13.214	2.280	1.152	0.000	0.000	0.000	0.000	27.453	0.511	6.871	0.436	7.843	0.429
Sample 5	8.668	13.699	0.972	1.683	0.000	0.000	0.000	0.000	32.609	0.599	62.430	1.714	63.541	1.716
Sample 6	44.459	8.821	0.039	0.068	0.000	0.000	0.000	0.000	52.566	0.412	148.211	3.692	149.964	3.682
Sample 7	35.341	10.427	5.461	0.634	2.136	0.255	0.000	0.000	10.995	0.385	33.266	0.857	34.196	0.875
Sample 8	12.339	13.924	11.998	1.711	0.000	0.000	0.000	0.000	27.285	0.632	20.419	0.363	21.629	0.368
Sample 9	8.461	11.275	0.509	0.882	0.000	0.000	0.000	0.000	11.512	0.163	9.904	0.255	10.301	0.251
Sample 10	5.641	7.904	0.000	0.000	0.000	0.000	0.000	0.000	26.596	0.340	4.953	0.276	5.840	0.282

DMP, dimethyl phthalate; DEP, diethyl phthalate; DBP, dibutyl phthalate; BBP, benzyl butyl phthalate; DEHA, di-ethylhexyl adipate; DEHP, di-(2-ethylhexyl) phthalate. Total represents the combined DEHP equivalent concentration for each sample.
